# Apamin Boosting of Synaptic Potentials in Ca_V_2.3 R-Type Ca^2+^ Channel Null Mice

**DOI:** 10.1371/journal.pone.0139332

**Published:** 2015-09-29

**Authors:** Kang Wang, Melissa H. Kelley, Wendy W. Wu, John P. Adelman, James Maylie

**Affiliations:** 1 Vollum Institute, Oregon Health & Science University, Portland, Oregon 97239, United States of America; 2 Department of Obstetrics and Gynecology, Oregon Health & Science University, Portland, Oregon 97239, United States of America; University of California, Berkeley, UNITED STATES

## Abstract

SK2- and K_V_4.2-containing K^+^ channels modulate evoked synaptic potentials in CA1 pyramidal neurons. Each is coupled to a distinct Ca^2+^ source that provides Ca^2+^-dependent feedback regulation to limit AMPA receptor (AMPAR)- and NMDA receptor (NMDAR)-mediated postsynaptic depolarization. SK2-containing channels are activated by Ca^2+^ entry through NMDARs, whereas K_V_4.2-containing channel availability is increased by Ca^2+^ entry through SNX-482 (SNX) sensitive Ca_V_2.3 R-type Ca^2+^ channels. Recent studies have challenged the functional coupling between NMDARs and SK2-containing channels, suggesting that synaptic SK2-containing channels are instead activated by Ca^2+^ entry through R-type Ca^2+^ channels. Furthermore, SNX has been implicated to have off target affects, which would challenge the proposed coupling between R-type Ca^2+^ channels and K_V_4.2-containing K^+^ channels. To reconcile these conflicting results, we evaluated the effect of SK channel blocker apamin and R-type Ca^2+^ channel blocker SNX on evoked excitatory postsynaptic potentials (EPSPs) in CA1 pyramidal neurons from Ca_V_2.3 null mice. The results show that in the absence of Ca_V_2.3 channels, apamin application still boosted EPSPs. The boosting effect of Ca_V_2.3 channel blockers on EPSPs observed in neurons from wild type mice was not observed in neurons from Ca_V_2.3 null mice. These data are consistent with a model in which SK2-containing channels are functionally coupled to NMDARs and K_V_4.2-containing channels to Ca_V_2.3 channels to provide negative feedback regulation of EPSPs in the spines of CA1 pyramidal neurons.

## Introduction

On hippocampal CA1 pyramidal neurons, dendritic spines are specialized membrane compartments that protrude from the dendrites and house proteins that mediate and shape excitatory postsynaptic responses[1]. Even within the small spine volume (~0.05 fL)[2], synaptic proteins are organized into discrete, functional domains. The postsynaptic density (PSD) is an electron-dense structure that contains ionotropic glutamate receptors, AMPARs and NMDARs that mediate excitatory postsynaptic responses. SK2-containing channels are also localized in the PSD[3]. These channels are activated by synaptically evoked Ca^2+^ influx through NMDARs, and their repolarizing conductance reduces glutamate-evoked excitatory postsynaptic responses and Ca^2+^ transients within the spine head. Thus, blocking synaptic SK2-containing channels with apamin increased EPSPs and the associated spine Ca^2+^ transients, while blocking NMDARs occludes the effects of apamin[4,5]. Several classes of ion channels and receptors reside in the extrasynaptic domain of the spine head. Among them are K_V_4.2-containing K^+^ channels and Ca_V_2.3 R-type Ca^2+^ channels[6]. Previous experiments using glutamate uncaging onto individual spines or direct afferent stimulation have reached different conclusions about the role of R-type Ca^2+^ channels in regulating EPSPs in CA1 pyramidal neurons. While both sets of experiments showed that blocking R-type Ca^2+^ channels with SNX boosted EPSPs, the effects of SNX and apamin were mutually exclusive when spines were stimulated by glutamate uncaging, suggesting that SK2-containing channels are gated by Ca^2+^ influx through R-type Ca^2+^ channels[5]. In contrast, the boosting effects of SNX and apamin were additive when direct afferent stimulation was applied[7]. Subsequent work showed that the boosting effect of SNX on EPSPs induced by glutamate uncaging was lost in Ca_V_2.3 null mice[8]. However, a recent report showed that in addition to blocking R-type Ca^2+^ channels, SNX blocks A-type K^+^ currents in dissociated dopamine neurons from substantia nigra pars compacta and cloned K_V_4.3 channels were much more sensitive to SNX compared to K_V_4.2 channels[9]. Furthermore, in cerebellar stellate cells where T-type Ca^2+^ channels couple to A-type K^+^ currents SNX had no effect on A-type channel availability, nor in tsA-201 cells co-expressing R-type (Ca_V_2.3) channels with K_V_4.2[10]. Therefore, we used synaptic stimulations to evoke EPSPs from CA1 pyramidal neurons in slices from Ca_V_2.3 R-type null mice to determine whether in the absence of Ca_V_2.3 channels, apamin and SNX still boosted EPSPs.

## Materials and Methods

### Animal Handling and Slice Preparation

All procedures were approved in accordance with the guidelines of the Institutional Animal Care and Use Committee (IACUC) of the Oregon Health & Science University (IACUC: IS00002421). Hippocampal slices were prepared from 4–6 week-old Ca_V_2.3 null (Ca_V_2.3^-/-^, C57BL/6J background) and wild type mice (C57BL/6J background). Mice were anesthetized by isofluorane, rapidly decapitated, and brains removed and placed into ice-cold sucrose-aCSF of the following composition (equilibrated with 95%O_2_/5%CO_2_) [7]. Transverse hippocampal slices (300 μm) were cut with a Leica VT1200S and transferred into a holding chamber containing regular aCSF (in mM: 125 NaCl, 2.5 KCl, 21.5 NaHCO_3_, 1.25 NaH_2_PO_4_, 2.0 CaCl_2_, 1.0 MgCl_2_, 12 glucose) and equilibrated with 95%O_2_/5%CO_2_. Slices were incubated at 35°C for 30–45 min and then recovered at room temperature (22–24°C) for ≥1 hr before recordings were performed.

### Electrophysiology

CA1 pyramidal cells were visualized with infrared–differential interference contrast optics (Zeiss Axioskop 2FS, Zeiss Axio Examiner or Leica DM LFS). Whole-cell patch-clamp recordings were obtained from CA1 pyramidal cells using an Axopatch 1D (Molecular Devices, Sunnyvale, CA) interfaced to an ITC-16 analog-to-digital converter (Heka Instruments, Bellmore, NY), EPC 10 (Heka Instruments, Bellmore, NY) patch clamp amplifier or Multiclamp 700B interfaced to a Digidata 1440A (Molecular Devices, Sunnyvale, CA). Data were transferred to a computer using Patchmaster software (Heka Instruments, Bellmore, NY) or pClamp10 software (Molecular Devices, Sunnyvale, CA). Patch pipettes (open pipette resistance, 2.5–3.5 MΩ) for EPSP recordings were filled with either a K-gluconate internal solution containing (in mM) 133 K-gluconate, 4 KCl, 4 NaCl, 2 MgCl_2_, 10 HEPES, 4 MgATP, 0.3 Na_3_GTP, 10 K-phosphocreatine (pH 7.3). EPSPs were recorded in whole-cell current-clamp mode and voltages were not corrected for a junction potential of -13 mV. All recordings used cells with a resting membrane potential less than -50 mV and a stable input resistance that did not change by more than 20%. Cells were biased to -65 mV and the input resistance was determined from a 25-pA hyperpolarizing current injection pulse given 500 ms after each synaptically evoked EPSP. There was not obvious difference in resting membrane properties in CA1 neurons from Ca_V_2.3^-/-^ compared to WT (average input resistance = 188.5 ± 6.3 MΩ (n = 55) in Ca_V_2.3^-/-^ and 201.4 ± 8.6 (n = 33) in WT; average bias current = -73.3 ± 7.1 pA (n = 55) in Ca_V_2.3^-/-^ and -76.5 ± 7.5 pA (n = 33) for WT).

### Synaptic stimulation

CA3 axons in the stratum radiatum were stimulated using capillary glass pipettes filled with aCSF, with a tip diameter of ~5 μm, connected to an Iso–Flex (A.M.P.I., Israel) or Digitimer DS3 (Automate Scientific, Berkeley, Ca) stimulus isolation unit. Stimulation electrodes were placed at ~100 μm from the soma and ~20 μm adjacent to the dendrite of the recorded cell. GABAergic blockers SR95531 (2 μM) and CGP55845 (1 μM) were present throughout the recordings to block GABA_A_ and GABA_B_ receptors, respectively. To prevent recurrent excitation in the CA3 region in the presence of GABAergic blockers, the CA3 region was cut away before recording. Subthreshold EPSPs were elicited by 100-μs current injections (20–30 μA) that were approximately one-third of the stimulus required for evoking an action potential. No obvious difference in stimulation amplitude was observed between slices from Ca_V_2.3^-/-^ and WT.

### Data analysis

Data were analyzed using Igor Pro (WaveMetrics, Lake Oswego, OR). Data are expressed as mean ± s.e.m. Paired t-tests or Wilcoxon-Mann-Whitney 2-sample rank test was used to determine significance; P < 0.05 was considered significant.

### Pharmacology

Apamin was purchased from Calbiochem; D-AP5, SR95531, and CGP55845 from Tocris Cookson; and SNX-482 from Peptide Institute.

## Results

**Fig 1:**
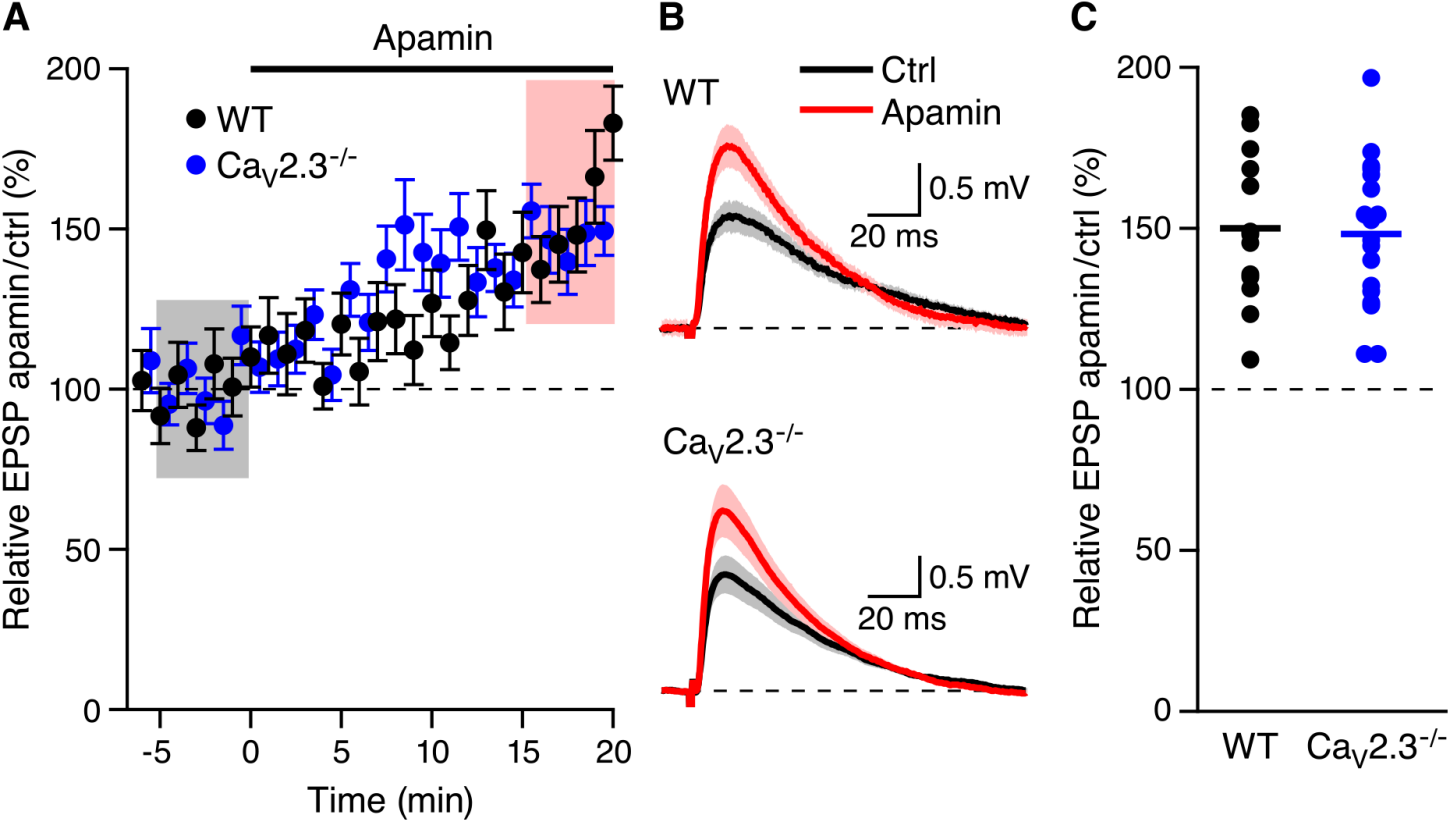
Apamin boosts EPSPs in Ca_V_2.3^-/-^ mice. (A) Time course of the normalized EPSP amplitude (mean ± s.e.m.) for baseline in control aCSF (Ctrl) and during wash-in of apamin (100 nM) as indicated above (n = 18) in Ca_V_2.3^-/-^ (blue symbols) and WT (black symbols) mice. (B) Average of 15 EPSPs taken from indicated shaded time points in aCSF (black) and 16–20 min after application of apamin (red); shaded areas are mean ± s.e.m. (C) Scatter plot of relative ESPS peak compared to baseline (Ctrl) from the individual slices in panel A for Ca_V_2.3^-/-^ (blue symbols) and WT (black symbols). Horizontal bar reflects mean response.

To determine whether blocking SK2-containing channels in spines lacking R-type Ca_V_2.3 Ca^2+^ channels boosts EPSPs, synaptic stimulations were delivered to the Shaffer collateral axons in the stratum radiatum in freshly prepared hippocampal slices from Ca_V_2.3 null mice, and EPSPs were measured from individual CA1 pyramidal neurons. After establishing a stable baseline, apamin (100 nM) was added to the bath solution. As shown in Fig 1A and 1B, apamin application boosted EPSPs (148.2 ± 5.4%, n = 18, P < 0.001). The increase in EPSP amplitude by apamin in mice lacking R-type Ca^2+^ channels was not different (p = 0.82) than the boosting effect of apamin in WT mice (158.2 ± 7.3%, n = 13, p < 0.001) (Fig 1C). These results indicate that Ca^2+^ influx through Ca_V_2.3 channels is not necessary to activate synaptic SK2-containing channels.

**Fig 2:**
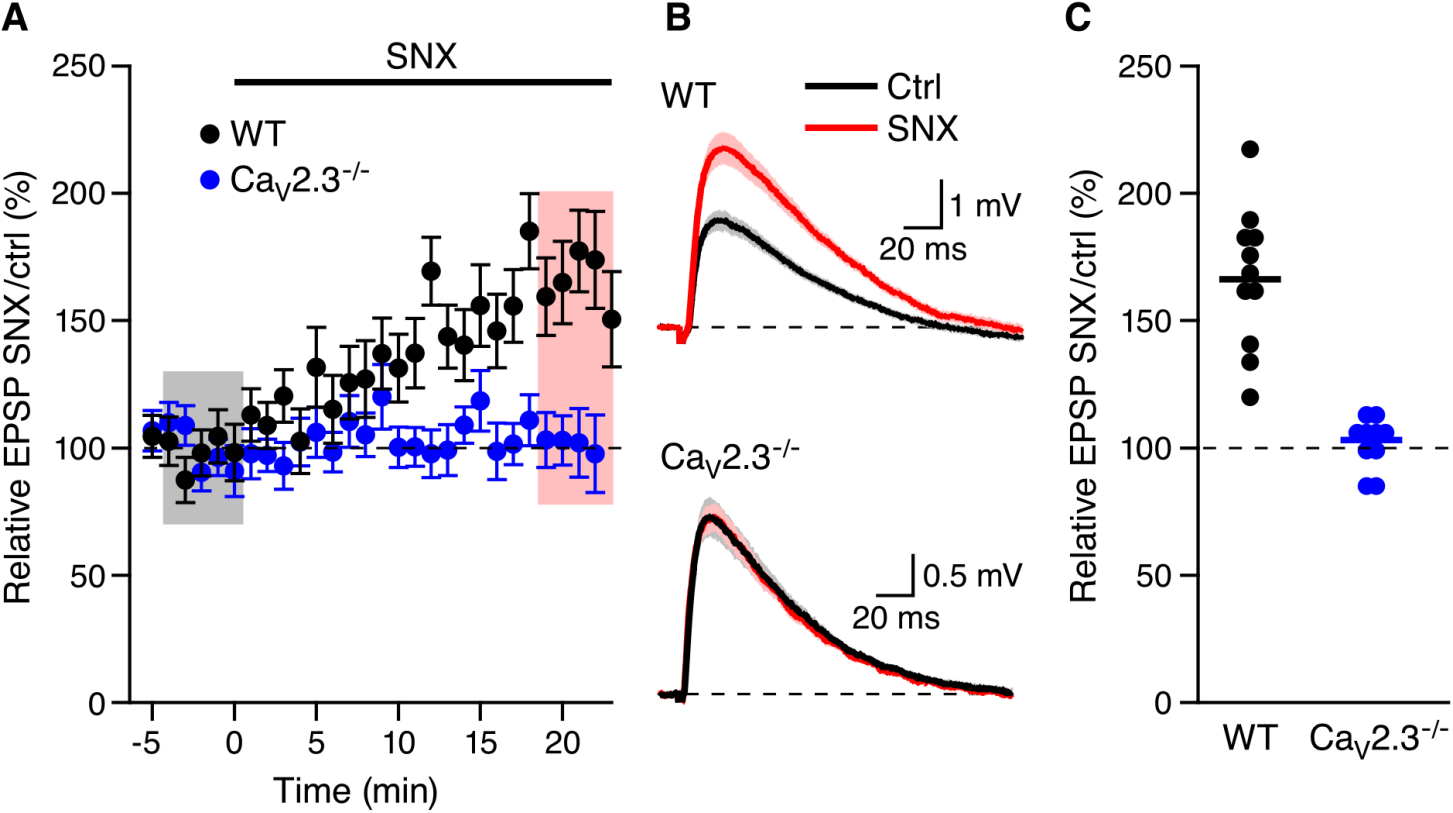
Boosting of EPSPs by SNX requires Ca_V_2.3 R-type Ca^2+^ channels. (A) Time course of the normalized EPSP amplitude (mean ± s.e.m.) for baseline in control aCSF (Ctrl) and during wash-in of SNX (300 nM) as indicated above in Ca_V_2.3^-/-^ (blue symbols) and WT (black symbols) mice. (B) Average of 15 EPSPs taken from indicated shaded time points in aCSF (black) and 19–23 min after application of SNX (red); shaded areas are mean ± s.e.m. (C) Scatter plot of relative ESPS peak compared to baseline (Ctrl) from the individual slices in panel A for Ca_V_2.3^-/-^ (blue symbols) and WT (black symbols). Horizontal bar reflects mean response.

**Fig 3:**
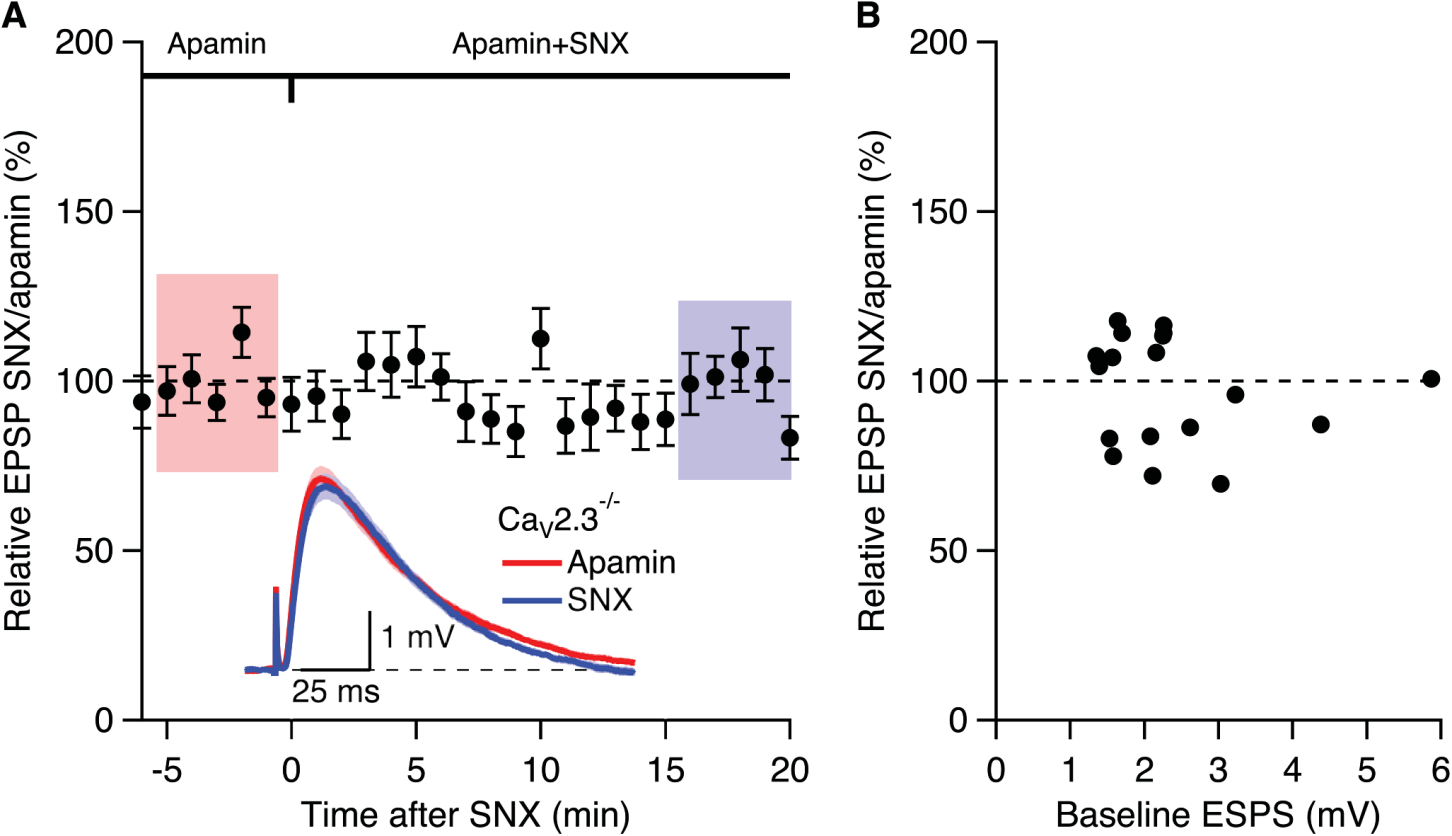
Boosting of EPSPs by SNX in the presence of apamin requires Ca_V_2.3 R-type Ca^2+^ channels. (A) Time course of the normalized EPSP amplitude (mean ± s.e.m.) for baseline in apamin (100 nM) and during wash-in of SNX (300 nM) in the presence of apamin (n = 19). Inset shows the average of 15 EPSPs taken from indicated shaded time points in apamin and 15–20 min after co-application of SNX; shaded areas are mean ± s.e.m. (B) Plot of relative EPSPs after SNX application in the presence of apamin versus the basline EPSP in apamin alone from all cells (n = 19).

In contrast, SNX (300 nM) that increased EPSPs in WT mice (166.2.0 ± 8.5%, n = 11, p < 0.001) did not affect EPSP amplitudes in mice lacking Ca_V_2.3 Ca^2+^ channels (103.1 ± 3.4%, n = 10) (Fig 2). Similarly, SNX (300 nM) that increased EPSPs in the presence of apamin in slices from WT mice (157.0 ± 7.8%, n = 21, p < 0.0001; see Fig 1 in Wang et al., 2014 [7]) did not affect EPSP amplitudes in slices from mice lacking Ca_V_2.3 R-type Ca^2+^ channels pretreated with apamin for 20–30 min (98.9 ± 3.8%; n = 19) (Fig 3A). Previously we showed that, in WT slices the apamin- and SNX-induced increase of EPSP were independent of initial EPSP size[7,11]. Fig 3B shows that the relative EPSP of SNX/apamin was independent of initial EPSP size; Fisher’s r to z analysis of the EPSP increase by SNX in the presence of apamin compared to the initial EPSP size in apamin yielded no correlation. These results suggests that the SNX boosting of EPSPs in CA1 neurons require Ca_V_2.3 R-type Ca^2+^ channels

**Fig 4:**
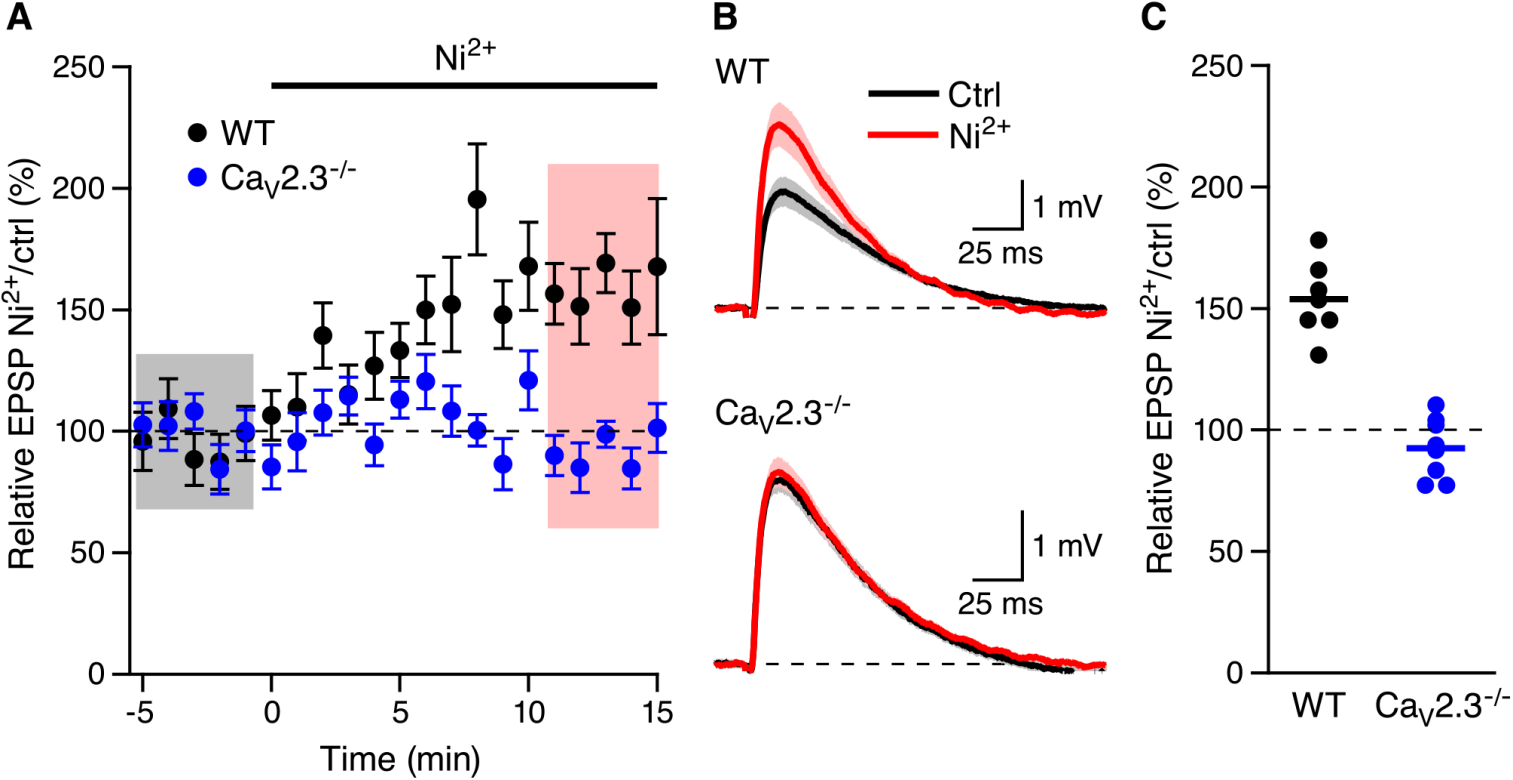
Boosting of EPSPs by Ni^2+^ requires Ca_V_2.3 R-type Ca^2+^ channels. (A) Time course of the normalized EPSP amplitude (mean ± s.e.m.) for baseline in control aCSF (Ctrl) and during wash-in of 100 μM Ni^2+^ in WT (black symbols, n = 7) and Ca_V_2.3^-/-^ mice (blue symbols, 8). (B) Average of 15 EPSPs taken from indicated shaded time points in aCSF (black) and 16–20 min after application of Ni^2+^ (red); shaded areas are mean ± s.e.m. (C) Scatter plot of relative ESPS peak compared to baseline (Ctrl) from the individual slices in panel A for Ca_V_2.3^-/-^ (blue symbols) and WT (black symbols). Horizontal bar reflects mean response.

SNX may have off target effects as recently reported[12]. Low concentrations of Ni^2+^ (100 μM) have also been used to block low voltage activated T-type and R-type Ca^2+^ channels [13–16]. As shown in Fig 4 Ni^2+^ (100 μM) increased EPSPs in WT mice (153.8 ± 5.9%, n = 7, P < 0.01) but not in Ca_V_2.3^-/-^ mice (92.4 ± 4.6%, n = 8, Fig 2). The sensitivity of Ca_V_2.3 Ca^2+^ channels to 100 μM Ni^2+^ is greatest at voltages less than -10 mV (>85% block)[17] a the voltage range that is likely not surpassed in dendritic spines during synaptic input[18]. Therefore, these results indicate that Ca_V_2.3 R-type Ca^2+^ channels are necessary for the boosting of EPSPs by SNX and Ni^2+^ in CA1 pyramidal neurons.

## Discussion

**Fig 5:**
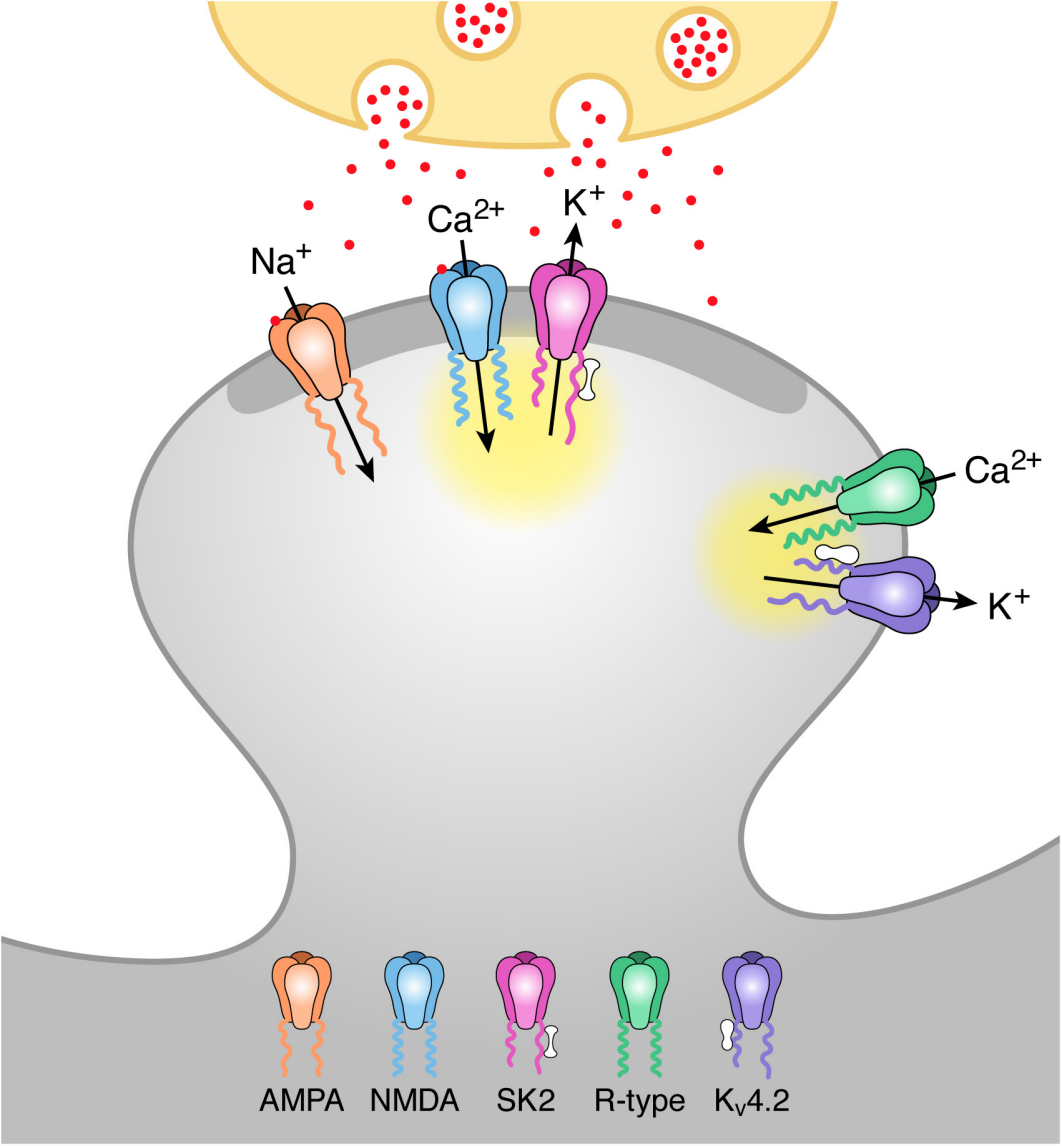
Model of activation of SK2 and K_V_4.2 containing channels by distinct Ca^2+^ microdomains during synaptic stimulation. Schaffer collateral stimulation releases glutamate (red particles) from the presynaptic terminal (ivory). Glutamate binding to AMPA and NMDA receptors in the postsynaptic density (PSD; dark grey) of the spine head depolarizes the spine membrane potential and releases voltage-dependent Mg^2+^ block from NMDA receptors allowing for Ca^2+^ influx during the EPSP. This Ca^2+^ activates closely associated SK2 channels via binding to calmodulin (barbell structure) bound to C-terminus of SK2 subunits. Spine depolarization also activates R-type Ca^2+^ channels located extrasynaptically that are close to K_V_4.2-containing K^+^ channels. Ca^2+^ entering through R-type channels binds to KChIPs (peanut structure) associated with K_V_4.2 channels shifting the voltage-dependence of availability to more negative potentials and allowing for K_V_4.2 activation during an EPSP. The yellow clouds represent the microdomain for each Ca^2+^ source.

These results show that in the absence of Ca_V_2.3 R-type Ca^2+^ channels, blocking SK2-containing channels with apamin boosts synaptic responses. Consistently, in these mice, we also found that SNX or Ni^2+^ provides no additional increase to EPSPs. These findings are consistent with synaptically evoked Ca^2+^ entry through NMDARs gating the synaptic SK2-containing channels[4]. They also support previous conclusions that Ca^2+^ influx through R-type Ca^2+^ channels binds to KChIPs to increase availability of K_V_4.2-containing A-type K^+^ channels, and blocking R-type Ca^2+^ channels with SNX or Ni^2+^ boosted synaptic potentials by decreasing availability of the repolarizing A-type K^+^ current[7]. Consistent with this, K_V_4.2 and Ca_V_2.3 proteins have been localized to the extrasynaptic region in CA1 spines[6,19]. This implies distinct Ca^2+^ signaling domains within the spine head, one coupling Ca^2+^ influx through NMDARs to activate SK2-containing channels and another coupling Ca^2+^ influx through R-type Ca^2+^ channels that activates K_V_4.2-contianing channels via KChIPs (Fig 5).

A recent report revealed that in addition to blocking R-type Ca^2+^ channels, SNX also blocks K_V_4-containing channels[9]. The lack of effect of SNX and Ni^2+^ in CA1 pyramidal neurons of Ca_V_2.3 mice, and the previous findings using Ca_V_2.3 R-type Ca^2+^ channel null mice[8], support the conclusion that R-type channels are necessary for the effects SNX and Ni^2+^ in CA1 pyramidal neurons. However, the present results do not address whether SNX also blocks K_V_4.2-containing K^+^ channels, as they may not be available to participate in synaptic responses in the absence of Ca^2+^ influx through R-type Ca^2+^ channels in Ca_V_2.3 null mice. Importantly, the lack of effect of Ni^2+^ and SNX in Ca_V_2.3 null mice supports the model that Ca^2+^ influx through R-type channels in CA1 provides the Ca^2+^ source to modulate K_V_4.2-containing K^+^ channel availability via associated KChIPs[7]. This is in contrast to cerebellar granule and stellate cells in which T-type Ca^2+^ channels couple to A-type K^+^ currents and SNX had no effect A-type channel availability[10,20].

Based upon results obtained using glutamate uncaging onto single dendritic spines, a model has been proposed in which the NMDAR dependence of synaptic boosting by apamin was not directly due to Ca^2+^ influx through NMDARs activating SK2-containing channels. Rather that NMDAR activation provided a necessary component of depolarization that activated R-type Ca^2+^ channels, and they provided the Ca^2+^ to fuel SK2-containing channel activation[5]. The present results used synaptic stimulations and suggest alternate conclusions. It should be noted that there are several distinctions between these studies that may be very significant. First, Kv4.2 and SK2 expression change with age[21,22] and the ages of the animals employed are different, P 16–18 for the uncaging studies while we used 4–8 week old mice. Second, we cannot be precisely sure of the location of the spines that are stimulated while the uncaging studies used spines on first oblique branches within 100 μm of the soma. Third, we do not know the nature of the stimulated spines, and the uncaging studies chose mushroom type spines. Given these differences, and while we cannot rule out the possibility that in the absence of R-type Ca^2+^ channels, the Ca^2+^ signaling domain architecture in the spine head is compromised, the present results are more consistent with Ca^2+^ influx through NMDARs fueling SK2-containing channel activation, a model supported by immuno-electron microscopy that showed close anatomical localization of SK2 and NMDAR within the PSD[3].
